# ShinyGPA: An interactive visualization toolkit for investigating pleiotropic architecture using GWAS datasets

**DOI:** 10.1371/journal.pone.0190949

**Published:** 2018-01-08

**Authors:** Emma Kortemeier, Paula S. Ramos, Kelly J. Hunt, Hang J. Kim, Gary Hardiman, Dongjun Chung

**Affiliations:** 1 Department of Public Health Sciences, Medical University of South Carolina, Charleston, SC 29425, United States of America; 2 Department of Medicine, Medical University of South Carolina, Charleston, SC 29425, United States of America; 3 Department of Mathematical Sciences, University of Cinncinati, Cinncinati, OH 45221, United States of America; Universita degli Studi di Roma Tor Vergata, ITALY

## Abstract

In spite of accumulating evidence suggesting that different complex traits share a common risk basis, namely pleiotropy, effective investigation of pleiotropic architecture still remains challenging. In order to address this challenge, we developed ShinyGPA, an interactive and dynamic visualization toolkit to investigate pleiotropic structure. ShinyGPA requires only the summary statistics from genome-wide association studies (GWAS), which reduces the burden on researchers using this tool. ShinyGPA allows users to effectively investigate genetic relationships among phenotypes using a flexible low-dimensional visualization and an intuitive user interface. In addition, ShinyGPA provides joint association mapping functionality that can facilitate biological understanding of the pleiotropic architecture. We analyzed GWAS summary statistics for 12 phenotypes using ShinyGPA and obtained visualization results and joint association mapping results that are well supported by the literature. The visualization produced by ShinyGPA can also be used as a hypothesis generating tool for relationships between phenotypes, which might also be used to improve the design of future genetic studies. ShinyGPA is currently available at https://dongjunchung.github.io/GPA/.

## Introduction

During the last decade, there have been concrete demonstrations of pleiotropy, i.e., a common genetic basis shared between distinct phenotypes [1]. Moreover, it has been shown that statistical power can be significantly improved by leveraging pleiotropy [2, 3]. Hence, accurate estimation of pleiotropy and utilizing pleiotropy for genetic data analysis can potentially improve our knowledge about the genetic basis of various phenotypes and diseases. Traditionally, pleiotropy has often been evaluated by first identifying genetic variants associated with each trait at the genome-wide significance level and then checking overlap of identified genetic variants across traits. However, this approach can often result in biased pleitropy estimates because uncertainty in association mapping is not properly taken into account in this estimation. This is especially the case when there are a large number of associated genetic variants with small effect sizes [1, 4], which is called polygenecity, becuase these genetic variants might not be identified via traditional statistical tests based on genetic study for a single phenotype. As a result, information from a significant number of associated variants is actually ignored in this approach. Thus, there is an urgent need for a statistically rigorous approach for analyzing pleiotropy.

In order to address these limitations, multiple statistical approaches to analyze pleiotropy have been proposed, including the genetic analysis incorporating pleiotropy and annotation (GPA) framework [3], the degree of surprise (DS) approach [5], the conditional false discovery rate approach [2], a risk-score profiling method [6], and a correlation-based method [7], among others. However, most of these methods require researchers to *a priori* select traits that might be genetically correlated with each other and are jointly analyzed, which is not a straightforward task in practice. This problem becomes even more challenging if the pleiotropic architecture among a large number of phenotypes is of interest. Furthermore, most of these tools also require to use command lines and/or write a code to run analyses of interest and this can become additional burden for the researchers.

In order to address these challenges, in this paper, we propose ShinyGPA which provides a flexible and dynamic visualization of pleiotropic architecture using an intuitive user interface, along with the joint association mapping functionality that allows biological understanding of the pleiotropic architecture. Notably, ShinyGPA requires that researchers provide only the summary statistics (genotype-phenotype association *p*-values) from genome-wide association studies (GWAS). This reduces researchers’ burden significantly and also allows wider application of this tool because summary statistics are often publicly available for various genetic studies. We believe that ShinyGPA will not only allow more effective analyses of genetic studies, but also potentially improve the design of future genetic studies.

## Materials and methods

**Fig 1:**
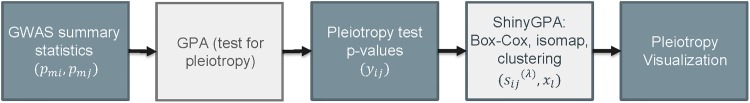
Summary of work flow for the ShinyGPA visualization approach.

Fig 1 shows the workflow of the ShinyGPA framework. Specifically, starting from the summary statistics of multiple GWAS (*p*_*mi*_, *p*_*mj*_), the GPA algorithm is applied to each phenotype pair and generates a matrix of pleiotropy test *p*-values (*y*_*ij*_). The resulting matrix of *p*-values is used as an input for the ShinyGPA, which utilizes a Box-Cox distance transformation ($s_{ij}^{\left(\lambda \right)}$) with a low dimensional visualization using the isometric feature mapping (*x*_*l*_). The end product is a dynamic, flexible pleiotropy visualization.

### Pairwise pleiotropy test using the GPA algorithm

In order to evaluate the pleiotropy between each pair of phenotypes, we utilized the pleiotropy test in the GPA approach [3]. Here we provide a brief review of this approach. Let *p*_*mk*_ represent the association *p*-value for the *m*-th single nucleotide polymorphism (SNP) from the *k*-th GWAS. In the two phenotype case, let *p*_11_, *p*_21_, …, *p*_*m*1_, …, *p*_*M*1_ be the association *p*-values for *M* SNPs from the first GWAS. Similarly, let *p*_12_, *p*_22_, …, *p*_*m*2_, …, *p*_*M*2_ be the association *p*-values from the second GWAS. Association *p*-values can be obtained from a *χ*^2^-test or a logistic regression in the context of a case-control GWAS.

Next we define a latent variable **Z**_*m*_ = {*Z*_*m*00_, *Z*_*m*10_, *Z*_*m*01_, *Z*_*m*11_} to indicate the association between the *m*-th SNP and the two phenotypes of interest. Specifically, *Z*_*m*00_ = 1 denotes the case where the *m*-th SNP is not associated with either of the phenotypes, *Z*_*m*10_ denotes the case where the *m*-th SNP is associated with only the first phenotype, *Z*_*m*01_ denotes the case where the *m*-th SNP is associated with only the second phenotype, and lastly, *Z*_*m*11_ denotes the case where the *m*-th SNP is associated with both phenotypes of interest. Here we assume that a SNP can only be in one of these states.

Given **Z**_*m*_, we assume the following emission distributions for GWAS association *p*-values:

$$\begin{array}{ccc} \pi_{00} & = & Pr(Z_{m00}=1):\\ & & \left(p_{m1}|Z_{m00}=1\right)∼U\left[0,1\right],\\ & & \left(p_{m2}|Z_{m00}=1\right)∼U\left[0,1\right].\\ \pi_{10} & = & Pr(Z_{m10}=1):\\ & & \left(p_{m1}|Z_{m10}=1\right)∼Beta\left(\alpha_{1},1\right),\\ & & \left(p_{m2}|Z_{m10}=1\right)∼U\left[0,1\right].\\ \pi_{01} & = & Pr(Z_{m01}=1):\\ & & \left(p_{m1}|Z_{m01}=1\right)∼U\left[0,1\right],\\ & & \left(p_{m2}|Z_{m01}=1\right)∼Beta\left(\alpha_{2},1\right).\\ \pi_{11} & = & Pr(Z_{m11}=1):\\ & & \left(p_{m1}|Z_{m11}=1\right)∼Beta\left(\alpha_{1},1\right),\\ & & \left(p_{m2}|Z_{m11}=1\right)∼Beta\left(\alpha_{2},1\right), \end{array}$$

where 0 < *α*_1_ < 1 and 0 < *α*_2_ < 1. Here, the null *p*-values are assumed to follow the Uniform distribution based on the Inverse Transform Theorem. Beta distribution was used to model associated *p*-values because it has the same range as the Uniform distribution, i.e., [0, 1]. Specifically, *Beta*(*α*, 1), 0 < *α* < 1, was considered here because its density monotonically decreases from 0 to 1 and this ensures that a smaller *p*-value is more likely than a larger *p*-value when it is from an associated group. In addition, *Beta*(*α*, 1) allows us to easily control the mass around zero using a single parameter *α*. Specifically, the smaller *α* is, the more mass we have around zero. Hence, the smaller *α* is often estimated for genetic studies with stronger signals, e.g., those with a larger sample size. Finally, as *α* → 1, *Beta*(*α*, 1) becomes the Uniform distribution.

Then, a likelihood ratio test is used to evaluate pleiotropy based on the null hypothesis

$$\begin{array}{c} H_{0}:\pi_{11}=\left(\pi_{10}+\pi_{11}\right)\left(\pi_{01}+\pi_{11}\right). \end{array}$$

Essentially, this null hypothesis implies that the signals from two GWAS are independent of each other. Hence, under the alternative hypothesis, the signals from two GWAS are not randomly distributed but there is statistical significant sharing of the signals between two GWAS datasets. Finally, a pleiotropy p-value for the pair of *i*-th and *j*-th phenotypes (*y*_*ij*_) is obtained based on the asymptotic null distribution of a *χ*^2^ distribution with 1 degree of freedom. Note that this asymptotic null distribution might be accurate only for a large number of SNPs. However, in our setting, we often consider 200K ∼ 10M SNPs, which is sufficient to use an asymptotic distribution to evaluate the likelihood ratio test statistic.

### Box-Cox distance transformation

The pleiotropy test described in the previous section returns a *p*-value for each pair of phenotypes. In the ShinyGPA framework, this *p*-value is considered as a distance measure between phenotypes. Specifically, a smaller *p*-value indicates stronger evidence for pleiotropy among that pair of phenotypes, i.e., closer distance. However, it might not be optimal to use this distance measure directly for visualization purposes, because while *p*-values can range from 0 to 1, the information is not equally distributed over this range. Specifically, in terms of *p*-values in general, the important information is concentrated around zero, and thus we are more interested in the values close to zero, compared to those farther away from zero. Since most visualization and clustering techniques do not take into account this skewed distance structure, we modified the distance measure so that it can perform effectively with most popular visualization and clustering algorithms.

While the log10 transformation is often used in the literature to expand the information around zero (e.g., [8]), we found that it is too rigid for what we are trying to accomplish in the ShinyGPA framework. So, in order to create a more flexible visualization, we decided to use a Box-Cox transformation:

$$\begin{array}{c} s_{ij}^{\left(\lambda \right)}=\left\{ \begin{array}{cc} \frac{y_{ij}^{\lambda }-1}{\lambda } & \text{if}\;\lambda \neq 0,\\ \ln(y_{ij}) & \text{if}\;\lambda =0, \end{array}\right. \end{array}$$ (1)

where *y*_*ij*_ is the original pleiotropy test *p*-value for the pair of *i*-th and *j*-th phenotypes and *λ* is a tuning parameter. In the ShinyGPA framework, instead of using a specific *λ* value, we have left the choice of *λ* up to the user, which allows for a dynamic, zoom-in/zoom-out functionality. Fig A in S1 Text illustrates the consequence of different *λ* choice for the Box-Cox transformation. The plots have the original *p*-values (*y*_*ij*_) on the *x*-axis and the transformed *p*-values ($s_{ij}^{\left(\lambda \right)}$) on the *y*-axis. Thus, the diagonal line produced with *λ* = 1 represents no transformation. A curve above the diagonal represents expanding the information around zero and the higher the point of inflection of the curve the more the small *p*-values are being expanded. Hence, decreasing the *λ* parameter will create a zoomed-out, bird’s eye view visualization while increasing the *λ* parameter will create a zoomed-in, detailed look at the pleiotropic structures. Note that *λ* = 0 is equivalent to a log10 distance transformation.

### Low dimensional visualization

After applying a Box-Cox transformation to the pleiotropy distance matrix (*y*_*ij*_), we apply isometric feature mapping (isomap) [9] to map phenotypes onto a two dimensional space based on their pleiotropic architecture. We chose isomap over more classic multi-dimensional scaling (MDS) algorithms to avoid the coordinates flipping on successive iterations of the algorithm. The isomap algorithm works in three steps. First, a neighborhood graph *G* is constructed over all the data points by connecting points *i* and *j* if either they are closer than some defined *ϵ*, or if *i* is one of the *k* (pre-defined) nearest neighbors of *j*. Then, it sets edge lengths to *d*_*x*_(*i*, *j*). The next step is to compute the shortest paths. This is done by initializing *d*_*G*_(*i*, *j*) = *d*_*x*_(*i*, *j*) if *i* and *j* are linked by an edge, and set *d*_*G*_(*i*, *j*) = ∞ otherwise. Then for each value of *k*, replace all entries of *d*_*G*_(*i*, *j*) with *min*{*d*_*G*_(*i*, *j*), *d*_*G*_(*i*, *k*) + *d*_*G*_(*k*, *j*)}. The resulting matrix of final values *D*_*G*_ = {*d*_*G*_(*i*, *j*)} will contain the shortest path distances between all pairs of points in G. The third step is to apply classical MDS to *D*_*G*_. This constructs an embedding of the data in a P-dimensional space that best preserves the geometry. The coordinate vectors **x**_*i*_ for points in *X* are chosen to minimize the objective function:

$$\begin{array}{c} E={∥\tau \left(D_{G}\right)-\tau \left(D_{X}\right)∥}_{L^{2}}, \end{array}$$

where *D*_*X*_ indicates matrix of Euclidean distances {*d*_*X*_(*i*, *j*) = ‖**x**_*i*_ − **x**_*j*_‖} and ‖*A*‖_*L*^2^_ is the *L*^2^ matrix norm $\sqrt{\sum_{i,j}A_{i,j}^{2}}$. The *τ* operator converts distance to inner products. The global minimum of the objective function is achieved by setting the coordinates **x**_*i*_ to the top *d* eigenvectors of the matrix *τ*(*D*_*G*_). In this way, we are able to graph the phenotypic relationships in a two dimensional plot. We used the isomap() function in R with epsilon = 0.15 and ndim = 2 to implement the isomap algorithm. Note that we chose epsilon = 0.15 because this is the smallest value that does not introduce the error of fragmented data.

### Phenotype clustering

In order to further facilitate interpretation of the visualization results for pleiotropic architecture described above, we apply a clustering algorithm to the coordinates generated from isomap that are already Box-Cox transformed. In the ShinyGPA framework, the user is able to determine the number of clusters and choose different clustering algorithms, including k-means and hierarchical clustering. We used the kmeans() and hclust() functions in R to implement the k-means and hierarchical clustering algorithms, respectively.

### Joint association mapping

While the phenotype plot generated by ShinyGPA provides an intuitive visualization of pleiotropic architecture, it still does not provide direct interpretation about why we obtain certain visualization results. In order to address this issue, we incorporate joint association mapping for each pair of phenotypes, which allows SNP-level explanation of the visualization results. Specifically, we utilized the joint association mapping functionality of the GPA algorithm, which is based on the local false discovery rate (FDR) that a SNP is shared between two phenotypes,

$$\begin{array}{c} locfdr_{m}=1-Pr\left(Z_{m11}=1|p_{m1},p_{m2}\right). \end{array}$$

We used the assoc() function in the GPA package to implement the joint association mapping. This helps elucidate which SNPs are driving the visualization, causing the pleiotropic structure.

### User interface

We implemented the ShinyGPA framework using the Shiny technology (https://shiny.rstudio.com/), which provides a dynamic and interactive user interface. Specifically, the user is able to choose visualization characteristics like which phenotypes are plotted, the number of clusters the phenotypes are partitioned into, and how far zoomed in or zoomed out they want to view the data. After the user specifies a setting, the plot is automatically updated, making this tool ideal for exploring pleiotropic relationships within the data. The interactive nature of the app allows the user to interact with the data and see how different choices (clustering methods or *λ* value for example) affect the interpretation of the degree of pleiotropy present.

In order to attain the portability of ShinyGPA, we implemented ShinyGPA as an R function and incorporated it as a part of the R package GPA (https://dongjunchung.github.io/GPA/). Specifically, there are two main functions to run ShinyGPA, namely fitAll() and shinyGPA(). It takes the GWAS association *p*-value matrix, namely pmat, as an input, where its rows and columns correspond to SNPs and phenotypes, respectively. If their row and column names are provided, these functions extract them automatically and use them in the Shiny app for easier interpretation. First, the following one-line command generates all the intermediate results required for the visualization.

R> out <- fitAll(pmat)

Next, the following command line takes the output of fitAll() as an input and opens the Shiny app, which will be illustrated in detail in the following subsections.

R> shinyGPA(out)

### Plot tab: Visualization of phenotype map

**Fig 2:**
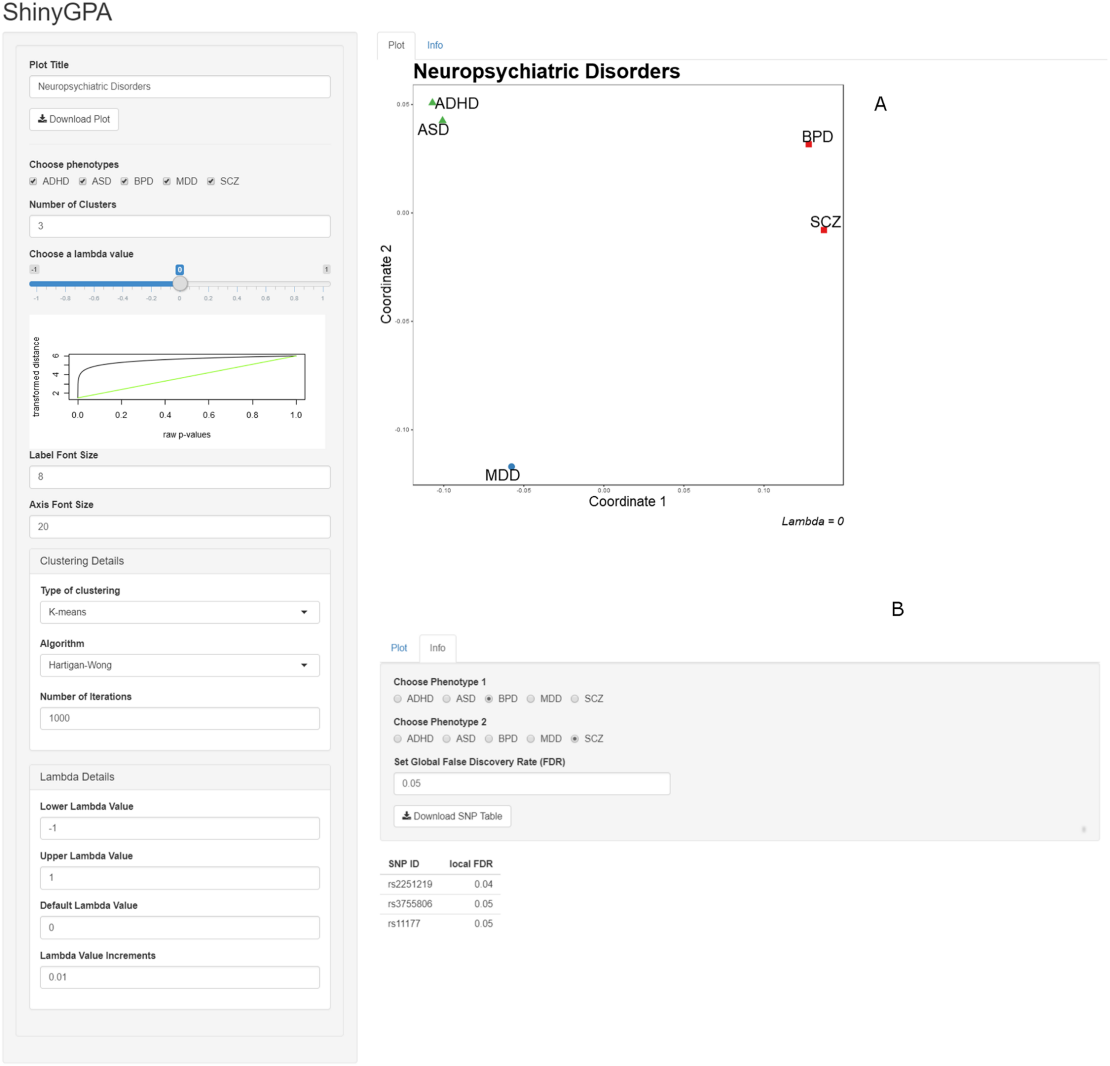
ShinyGPA screenshot with the “Plot” tab open (A) and the “Info” tab open (B).

Fig 2A shows the layout of ShinyGPA, where the “Plot” tab is open by default. On the left side of the screen, the user input options begin with the plot title input field. Below the title input is the download button. This allows the user to download the main phenotype plot as a Portable Document Format (PDF) file, which is automatically named *YourPlotTitle*-Shinyplot.pdf. Next, there is a group of phenotype check boxes. By unchecking the boxes, the user is able to customize the pleitropy visualization to the specific phenotypes of interest. This feature is helpful for exploring pleiotropic relationships as phenotypes can easily be removed and added back in with a simple click. Next, the user is able to choose the number of clusters applied to the phenotypes. The phenotypes are clustered by shape as well as by color, so that the results are clear even if the plot is eventually printed in gray scale. Below the clustering options, there are the distance transformation controls. This includes the *λ* slider bar which provides zoom-in / zoom-out functionality as well as a small plot to show how the *λ* choice is transforming the *p*-values. Near the bottom of the control panel are fields that adjust the font size of both the phenotype labels on the graph and the coordinate labels. This allows the user to optimize the visualization results so that they can be easily utilized for research article and grant proposal purposes. Lastly, at the bottom of the input options, there are fields to customize the *λ* slider bar itself, as well as the clustering algorithm if the user desires.

### Info tab: Joint association mapping

Next, if the user clicks the “Info” tab, it will open the user interface for joint association mapping as seen in Fig 2B. Here, the user can find more information on the specific SNPs that are driving the visualization. The user is able to select the two phenotypes of specific interest as well as the global false discovery rate (FDR) and the joint association mapping results are automatically updated. The resulting table shows the list of SNPs that are associated with the specified pair of phenotypes at the specified global FDR level, along with their local FDR values. The user can also download the result table as a Microsoft Excel comma separated values (CSV) file.

### Data description

We considered the summary statistics from 12 GWAS datasets as input for ShinyGPA, including neuropsychiatric disorders: attention deficit-hyperactivity disorder (ADHD), autism spectrum disorder (ASD), bipolar disorder (BPD), major depressive disorder (MDD), schizophrenia (SCZ) [7, 10]; autoimmune diseases: Crohn’s disease (CD) [11], ulcerative colitis (UC) [12], rheumatiod arthritis (RA) [13]; lipid-related phenotypes: high-density lipoprotein (HDL) [14], type 2 diabetes (T2D) [15]; cardiovascular phenotypes: coronary artery disease (CAD) [16], and systolic blood pressure (SBP) [17]. More details about these GWAS datasets can be found in Table A in S1 Text.

## Results

### Simulation studies

**Fig 3:**
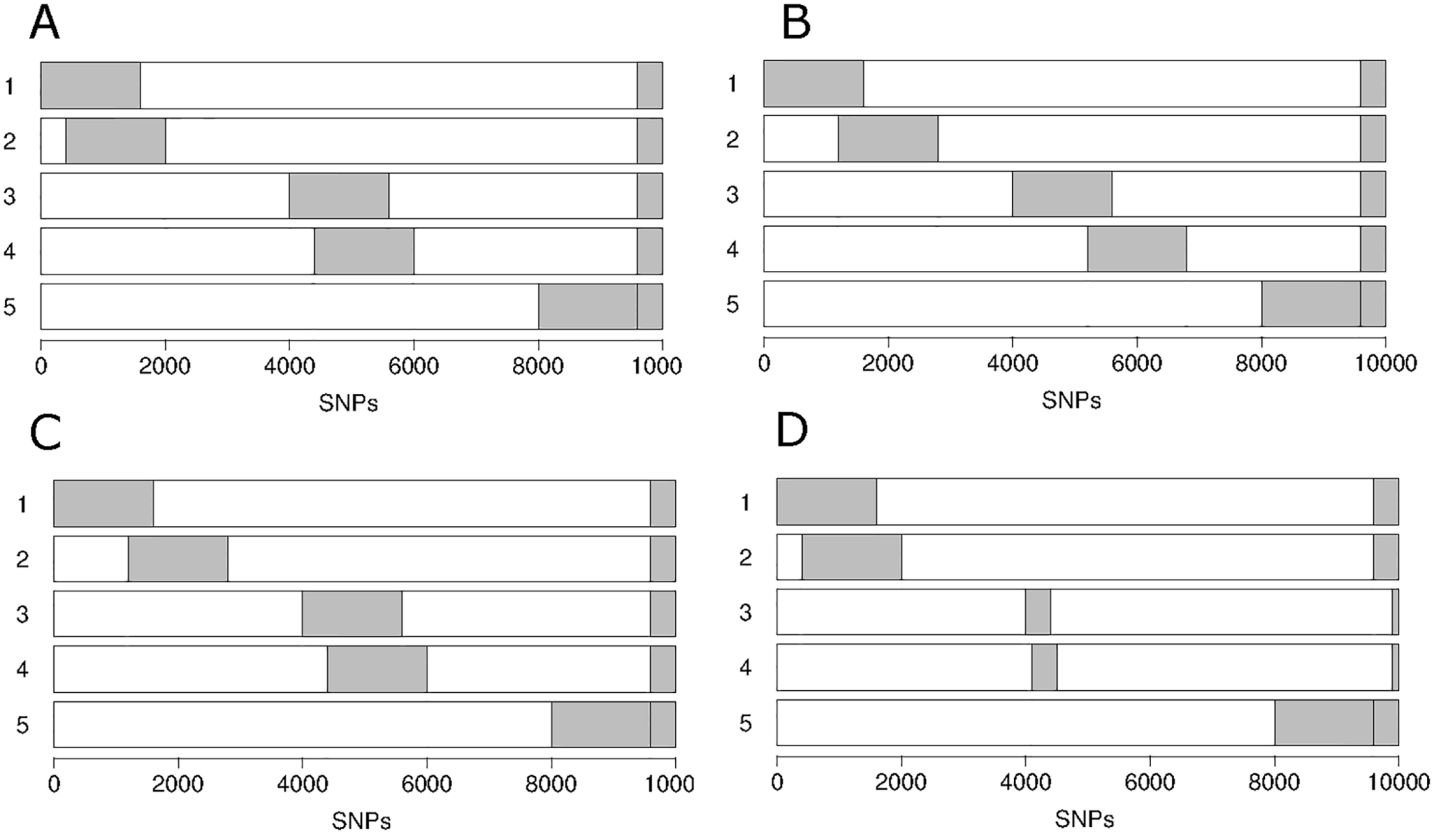
Simulation settings. (A) 20% associated SNPs for each phenotype and 75% extra overlaps between GWAS_1 and GWAS_2 and between GWAS_3 and GWAS_4; (B) 20% associated SNPs and 25% extra overlaps; (C) 20% associated SNPs with varying extra overlaps (25% between GWAS_1 and GWAS_2 and 75% between GWAS_3 and GWAS_4); and (D) Varying proportion of associated SNPs (20% for GWAS_1 and GWAS_2 and 5% for GWAS_3 and GWAS_4) with 75% extra overlaps.

**Fig 4:**
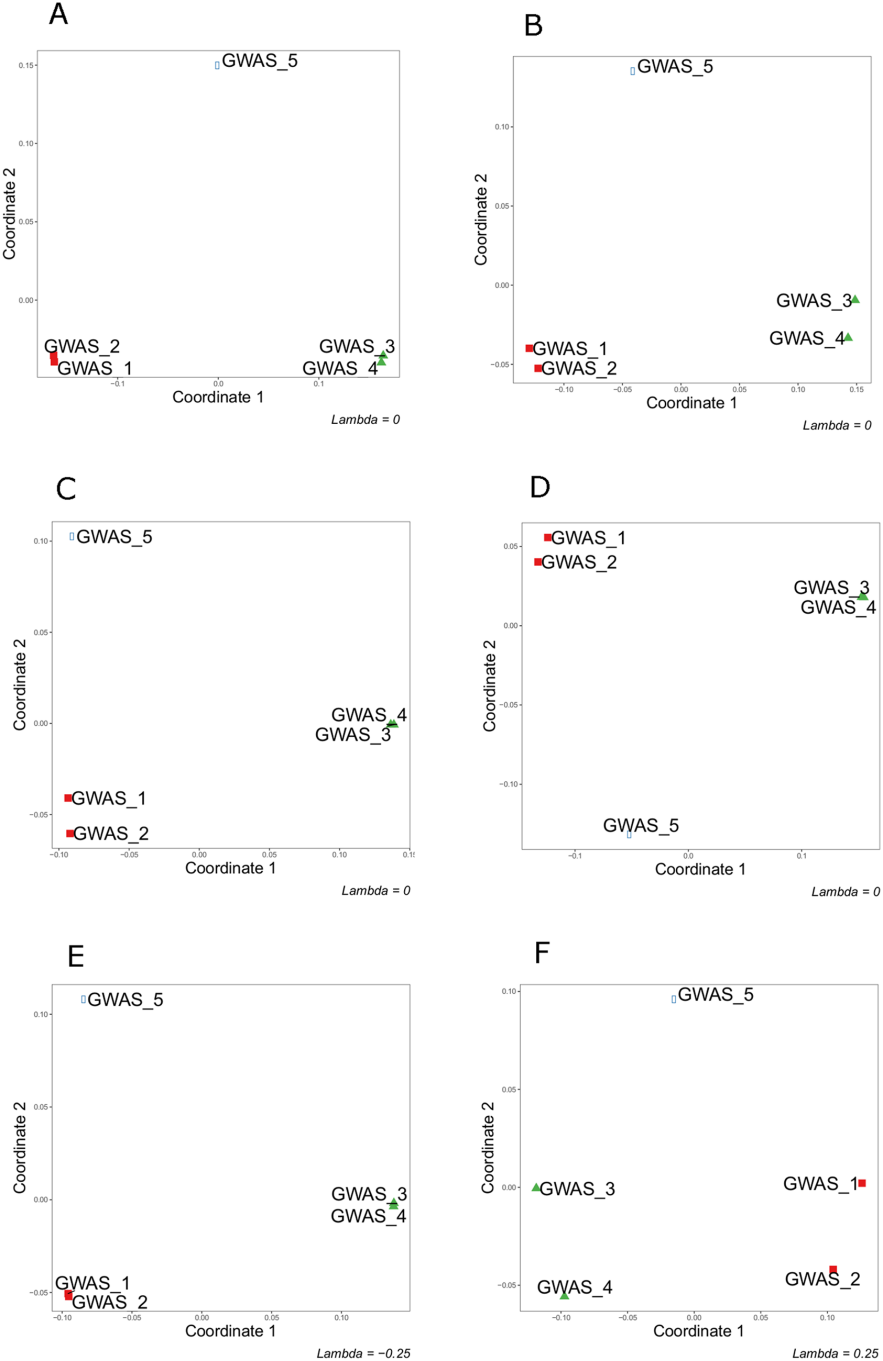
ShinyGPA plots. (A)—(D) correspond to the simulation settings of Fig 3A-3D when *λ* = 0. (E) and (F) show the plots of Fig 3B for *λ* = −0.25 and *λ* = 0.25, respectively.

We first evaluated ShinyGPA using simulation studies. First, we considered five phenotypes such that each phenotype has 20% associated SNPs and 80% background SNPs. We set the associated SNPs of all five phenotypes to have a 4% overlap, which is what is expected by chance assuming no pleiotropy (0.2 ∗ 0.2 = 0.04). In addition, we set the pair of phenotype 1 (GWAS_1) and phenotype 2 (GWAS_2) and the pair of phenotype 3 (GWAS_3) and phenotype 4 (GWAS_4) to have 75% extra overlap. As a negative control, phenotype 5 (GWAS_5) was set to be independent and not have any extra overlap with any other phenotype. Fig 3A provides a visual for the simulation setting, specifically how we designed the five phenotypes to overlap. Using the GPA generative model [3], the *p*-values of the associated SNPs were generated from *Beta*(0.4, 1) while the *p*-values of the background SNPs were generated from a uniform distribution. Fig 4A provides the corresponding ShinyGPA plot. As expected, GWAS_1 is clustered with GWAS_2 and GWAS_3 makes a cluster with GWAS_4 while GWAS_5 is located away from all of them.

Second, in order to see the effects of varying degrees of pleiotropy, we considered various degrees of extra overlap among phenotypes. Specifically, when we make the degree of extra overlap lower as 25% instead of 75% (Fig 3B), the distance between the points on the plot increases for a fixed *λ* (Fig 4B). Next, we set GWAS_3 and GWAS_4 to have higher degree of extra overlap (75%) compared to GWAS_1 and GWAS_2 (25%), as depicted in Fig 3C. In this case of the mixed overlap, ShinyGPA correctly plots GWAS_3 and GWAS_4 nearer to each other than GWAS_1 and GWAS_2, with GWAS_5 being furthest away of all (Fig 4C). Third, one of the key features of the proposed ShinyGPA framework is that it is not affected by the proportion of associated SNPs as long as the degree of pleiotropy remains the same. In order to confirm this property, we made each of GWAS_3 and GWAS_4 has only 5% associated SNPs while the degree of extra overlap between GWAS_3 and GWAS_4 remains as 75% (Fig 3D) as Fig 3A. As expected, the resulting phenotype map (Fig 4D) essentially remains similar to Fig 4A.

Finally, in order to evaluate the visualization flexibility provided by the *λ* parameter, we generated phenotype maps for the simulation data of Fig 3B (extra 25% overlap) but with varying *λ* values. When *λ* = 0 (Fig 4B), we have a classic log10 transformation. When *λ* is negative (Fig 4E), it acts as a “zoom-out” transformation, allowing the overall relationships among the phenotypes to be visualized. On the other hand, when *λ* is positive (Fig 4F), it acts as a “zoom-in” transformation, allowing a more detailed look into the pleiotropic relationships. In summary, this simulation study showed that ShinyGPA can potentially recover true genetic relationships among phenotypes and provide desirable flexibility using the *λ* parameter.

### Joint analysis of GWAS datasets for 12 phenotypes

**Fig 5:**
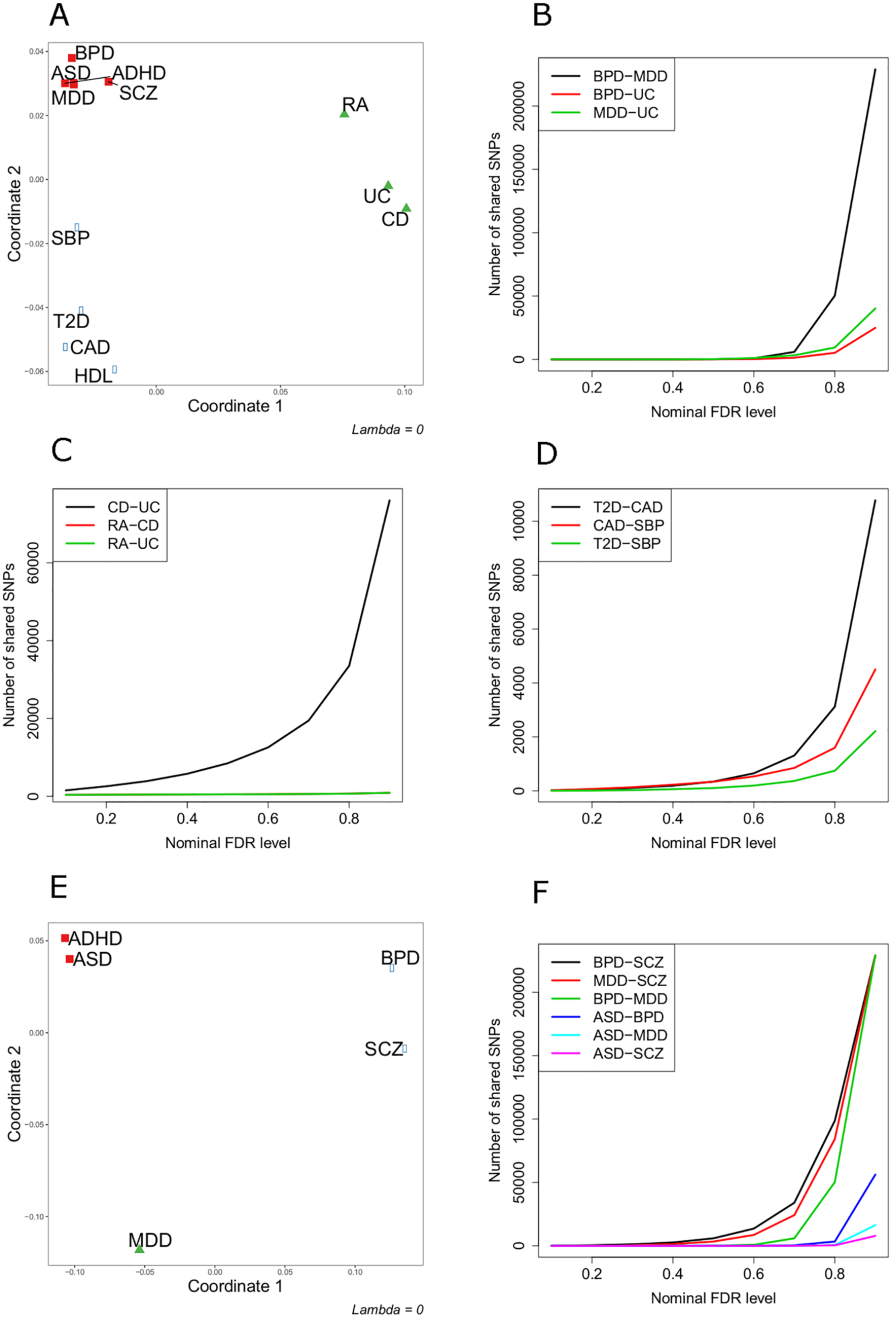
Joint analysis of GWAS datasets for 12 phenotypes. (A) ShinyGPA plot for the 12 phenotypes. (B—D) Numbers of SNPs shared between various pairs of phenotypes (B: BPD-MDD-UC; C: RA-UC-CD, D: T2D-CAD-SBP) under various nominal local FDR levels. (E) ShinyGPA plot for the five neuropsychiatric disorders. (F) Numbers of SNPs shared between each pair of neuropsychiatric disorders under different nominal local FDR levels. ADHD is excluded in this plot because too few SNPs were identified to be associated with ADHD regardless of FDR levels.

Fig 5A shows the ShinyGPA visualization of the 12 enumerated phenotypes, with 3 clusters and *λ* = 0 defined. We see that the neuropsychiatric phenotypes make a cluster, the autoimmune diseases make a cluster, and the lipid-related and cardiovascular phenotypes make a third cluster. Specifically, the cluster made of up RA, UC, and CD is supported in the literature as they are all three autoimmune diseases [18]. There is also evidence of pleiotropy among the five neuropsychiatric disorders, which supports the cluster made up of ASD, MDD, ADHD, SCZ, and BPD [2, 6, 7, 19]. The pleiotropy between T2D and CAD has been reported in multiple studies [20–25]. When we increase the number of clusters to 5 and keep *λ* = 0 (Fig B2 in S1 Text), we see that UC and CD make a cluster, RA stands alone, the five neuropsychiatric phenotypes make the third cluster, SBP has its own cluster, and the rest of the lipid-related and cardiovascular phenotypes make the fifth cluster. In comparing the plots with different *λ* values, we see that when *λ* is very negative (-0.04) (Fig C1 in S1 Text), all the phenotypes converge to one point, except the three autoimmune diseases. When *λ* = −0.02 (Fig C2 in S1 Text) we see the neuropsychiatric disease getting closer to each other. Lastly, when *λ* is positive (0.02) (Fig C3 in S1 Text) we see a spreading out of the phenotypes, allowing a closer look into each group.

In order to further validate this visualization result, we evaluated whether more SNPs are shared between pairs of phenotypes that are located more closely in the map. Specifically, for each pair of phenotypes, we identified SNPs shared between two phenotypes using the joint association mapping functionality of the R package GPA (function assoc() with the argument pattern = “11”). When the set of phenotypes BPD, MDD, and UC are considered (Fig 5B), BPD and MDD that are located very close from each other share a larger number of SNPs compared to those between BPD-UC or MDD-UC. Among the three autoimmune diseases (RA, UC, and CD), two inflammatory bowel diseases, UC and CD, again share a much larger number of SNPs than UC-RA and CD-RA (Fig 5C). Finally, in the phenotype map, T2D and CAD are located closer from each other compared to SBP and HDL (Fig 5A). Consistently with the visualization result, T2D and CAD actually also share a larger number of SNPs compared to CAD-SBP and T2D-SBP (Fig 5D).

Next, when we investigate only the five neuropsychiatric disorders together (Fig 5E), BPD and SCZ make one cluster, ADHD and ASD make another cluster, and MDD is off by itself. Note that the strong pleiotropy between BPD and SCZ is well supported by the literature [6]. We again checked numbers of SNPs shared between each pair of these neuropsychiatric disorders (Fig 5F; we did not include ADHD in this plot because the number of SNPs associated with ADHD is too small for this analysis). As expected, BPD and SCZ share the largest number of SNPs among all the possible pairs, followed by pairs involving MDD and then by pairs involving ASD. Also note that SCZ that is located closer to MDD also shares more SNPs with MDD, compared to BPD that is located farther from MDD in this visualization map. We further checked the SNPs that are shared between BPD and SCZ in order to determine whether the SNP sharing is biologically meaningful. We found that the top ranking SNPs include rs9951150 (local FDR = 0.042) and rs7096169 (local FDR = 0.057), which were previously reported to be potentially shared among five psychiatric disorders [10].

### SNPs shared among autoimmune diseases

Given the closeness among UC, CD, and RA in our visualization map, we sought to understand the potential biological mechanisms explaining the SNPs identified as shared between each pair of phenotypes. Specifically, we ranked the SNPs identified as shared between each pair of phenotypes using the GPA algorithm according to their nominal global FDR levels, and considered SNPs with a nominal global FDR level under 0.05 or 0.1. For the SNPs shared between 1) RA and CD, 2) RA and UC, and 3) CD and UC, we obtained their positions and reported GWAS associations from the NHGRI-EBI GWAS Catalog (https://www.ebi.ac.uk/gwas/) accessed on August 25th, 2017. The top ranked shared SNPs were then evaluated based on their location relative to current GWAS associations.

Top SNPs shared between RA and CD, as well as RA and UC or UC and CD, lie in regions well-known to be shared across multiple autoimmune diseases, such as the *HLA*, *IL23R*, *TNFAIP3*, and *IL2RA* [26]. RA and CD shared 576 SNPs with FDR < 0.1 and 497 SNPs with FDR < 0.05, with the majority of these (61% of all SNPs with FDR < 0.1, and 67% of all SNPs with FDR < 0.05) mapping to the extended major histocompatibility (MHC) region, also known as the human leukocyte antigen (HLA) complex. This region encompasses approximately 7.6 Mb and 421 annotated gene loci at 6p22.1-6p21.32 [27], and is in high linkage disequilibrium and strongly associated with all autoimmune diseases and many inflammatory traits. We observed top shared SNPs located in the HLA classical class III, class II, and class I regions (e.g., *NOTCH4*, *HLA-DR*, and *HLA-B*, respectively). Given its strong association signals and sharing among all autoimmune diseases, the sharing of SNPs between RA and CD is thus unsurprising.

Multiple strongly shared SNPs between RA and CD were also observed in the *MAGI3-PTPN22* region at 1p13.2 (31 SNPs with FDR < 0.1; 24 SNPs with FDR < 0.05). Top shared SNPs in this region are associated with multiple autoimmune diseases, including RA, CD, systemic lupus erythematosus (SLE), T1D, vitiligo and myasthenia gravis. Interestingly, other SNPs in this region have been associated with multiple other traits (e.g., leprosy, bacteremia, thyroid peroxidase antibody levels, and hypothyroidism), supporting the value of ShinyGPA to unveil novel pleiotropic relationships. The next strongest shared SNPs (FDR < 0.05) also map to shared immune loci. The top SNP near *CD40* (20q13.12) has been associated with IBD, CD and Kawasaki disease; this regions is associated with RA, multiple sclerosis, tonsillectomy, and chronic hepatitis B infection. Five SNPs near *TAGAP* (6q25.3) have been associated with IBD, CD, celiac disease and multiple sclerosis. This region’s association with lipoprotein (a) levels corroborates the value of ShinyGPA in unveiling novel pleiotropic effects. Two SNPs near *TNFAIP3* (6q23.3) have been reported as associated with celiac disease, with other SNPs in the region being associated with other autoimmune diseases (UC, SLE, RA, psoriasis, multiple sclerosis, Sjögren’s syndrome, primary sclerosing cholangitis). Finally, seven SNPs near *IL2RA* (10p15.1) have been reported as associated with RA, T1D, multiple sclerosis, alopecia areata, primary sclerosing cholangitis, and plasma t-tau levels, with other SNPs in the region being associated with several immune-related traits.

Similarly to the sharing between RA and CD, RA and UC shared 576 SNPs with FDR < 0.1 and 369 SNPs with FDR < 0.05, with the majority of these (60% of all SNPs with FDR < 0.1, and 76% of all SNPs with FDR < 0.05) mapping to the extended MHC region. Several top SNPs (FDR < 0.05) also map to the region adjacent to the classical HLA class II region (e.g., *IP6K3* (associated with educational attainment and phosphorus levels) and *BAK1* (associated with platelet count, chronic lymphocytic leukemia, and testicular germ cell tumor)). Strongly shared SNPs were observed in multiple regions, including near *TNFAIP3* (2 SNPs with FDR < 0.05), *CD40* (one SNP with FDR < 0.05), *MAGI3-PTPN22* (16 SNPs with FDR < 0.05), and *IL2RA* (6 SNPs with FDR < 0.05).

Since they are both chronic inflammatory bowel diseases (IBDs), CD and UC shared the most SNPs (1,556 with FDR < 0.1; 1,013 with FDR < 0.05). In contrast to the aforementioned SNPs shared between the IBDs and RA, only up to 7% of the top shared SNPs between the IBDs map to the HLA region. The most strongly associated SNPs map to the *IL23R* region at 1p31.3 (31 SNPs with FDR < 0.1, 23 SNPs with FDR < 0.05). These SNPs are associated with autoimmune diseases (CD, UC, psoriasis, Behçet’s disease, primary biliary cholangitis) as well as other traits (leprosy, linoleic acid levels). The sharing of this region extends to other autoimmune diseases (e.g., ankylosing spondylitis, psoriatic arthritis). The next strongest shared SNPs (six SNPs with FDR < 0.1) map near *NKX2-1* (10q24.2). In addition to their associations with CD and UC, the shared SNPs have also been associated with dental caries, and other SNPs in this region are associated with multiple other traits (e.g., red blood cell traits, blood protein levels, or colorectal cancer), supporting the value of ShinyGPA in discovering novel pleiotropic effects. One of the most explicit examples of the utility of ShinyGPA to uncover novel pleiotropic effects is that of the *MST1-CAMKV* region at 3p21.31; while the region is associated with CD, UC, primary sclerosing cholangitis, blood protein levels, and educational attainment, the top 21 shared SNPs (FDR < 0.1) are associated with two new traits: resting heart rate and age at first birth. Fifteen SNPs (FDR < 0.1) near *JAK2* (9p24) are associated with IBD, CD, UC, and several red blood cell traits; SLE, psoriatic arthritis and myeloproliferative neoplasms are other associations in the region. Thirteen SNPs near IL12B (5q33.3) are associated with multiple sclerosis, psoriasis, primary biliary cholangitis, while the region’s associations extend to other autoimmune diseases (CD, UC, psoriatic arthritis, and ankylosing spondylitis). Ten shared SNPs near *TNFRSF6B* (20q13.33) are associated with IBD, CD, glioma and atopic dermatitis; this regions is associated with UC, glioblastoma, prostate cancer and lung function. Finally, 28 top shared SNPs near *PTGER4* (5p13.1) are associated with CD, multiple sclerosis, and self-reported allergy, with other SNPs in the region being associated with immune-related traits (UC, psoriasis, selective IgA deficiency, ankylosing spondylitis, and blood protein levels).

### SNPs shared between type II diabetes and coronary artery disease

Elevated cardiovascular risk factors in pre-diabetic individuals [20–23], elevated cardiovascular risk prior to a clinical diagnosis of T2D in the Nurses’ Health Study [24], and elevated carotid artery intima-media thickness in pre-diabetic individuals in our Mexico City study [25], each suggest an atherogenic state prior to the onset of clinical diabetes that is consistent with a common etiology underlying T2D and cardiovascular disease. Moreover, diabetes is considered a CAD risk equivalent in that individuals with diabetes are at similar risk of having an incident CAD event as individuals who have had a prior myocardial infarction [28]. However, despite evidence of underlying common etiology between T2D and cardiovascular disease, the mechanistic link between the diseases remains poorly understood [29–31].

Examining the pleiotropic architecture underlying T2D and CAD may provide mechanistic insight into the common etiology of these diseases. Similar to what is described for the SNPs shared among autoimmune diseases, we selected the SNPs shared between T2D and CAD with a global FDR level under 0.05 and 0.1, and obtained their positions, as well as reported GWAS associations. The top ranked shared SNPs were then evaluated based on their location and reported association in the GWAS Catalog.

In our analysis, T2D and CAD shared 43 SNPs with FDR < 0.1 and 19 SNPs with a FDR < 0.05. Of the top 19 SNPs shared between T2D and CAD, the *insulin receptor substrate 1* (*IRS1*) region (2q36.3) is one of the most interesting, with the four shared top SNPs being associated with T2D, insulin resistance, hyperinsulinemia, tryglicerides, and HDL cholesterol levels. Other variants in this region have been associated with CAD, fasting insulin-related traits, adiposity, body fat percentage, adiponectin levels, body mass index, waist circumference, and waist-hip ratio. Collectively, these associations support the hypothesis that shared metabolic dysfunction mechanisms underlie the etiology of these traits.

Also revealing is the region of the non-coding RNA *CDKN2B-AS1* (9p21.3), which in addition to association with CAD and T2D, has eight top shared SNPs (FDR < 0.05) showing associations with glaucoma and multiple cancers, unveiling novel pleiotropy. The last cluster of three shared SNPs map to the *SNF8-GIP* region (17q21.32); although the shared SNPs have not been reported in GWAS, other SNPs in this region are associated with CAD and myocardial infarction. In summary, systemically examining pleiotropy between diseases believed to share common antecedents can be used as a hypothesis generating tool to identify potentially novel mechanistic targets of shared disease etiology.

## Discussion

Here we presented a novel approach to investigate pleiotropy, namely ShinyGPA, which provides a flexible and interactively dynamic visualization that has guidance for interpretation. Key features include a flexible distance transformation, an interactive experience, and a phenotype clustering to guide interpretation. In addition, ShinyGPA provides various options to improve user experience, such as a control of title and font sizes; a downloading function to save the visualization results as a PDF file; and the joint association mapping results as a CSV file, all of which can be useful for various publication purposes. In order to guarantee biologically meaningful results, we note that users need to confirm the following key assumptions made in ShinyGPA:

- It is critical to provide ShinyGPA a valid set of genotype-phenotype association *p*-values from upstream GWAS analyses. Specifically, in order to evaluate the pleiotropy, ShinyGPA utilizes the GPA model, which is based on a Beta-Uniform mixture model. For example, if population stratification and cryptic relatedness are not properly taken into account or incorrect genetic association models are used in the GWAS analysis step, the obtained *p*-values for null SNPs might not follow the Uniform distribution and this can affect accuracy of the phenotype map generated by ShinyGPA.
- As the ShinyGPA framework utilizes the GPA model to evaluate the pleiotropy for each pair of phenotypes, ShinyGPA outputs are affected by the assumptions made in the GPA model. Specifically, the GPA model assumes independence between SNPs and hence, the presence of linkage disequilibrium (LD) may affect the ShinyGPA output. For example, if there is a wide region with strong LD and a large number of SNPs within this LD region are shared between two phenotypes, the pleiotropy test *p*-value can become smaller for this pair of phenotypes. However, we believe that this issue will usually not distort the ShinyGPA output significantly because of the following reasons. First, in the original GPA paper, we investigated the impact of LD on the GPA approach using simulation studies and showed that while LD does impact the measure of pleiotropy, the GPA results are still robust to a moderate degree of LD [3]. Second, we note that ShinyGPA takes into account all possible combinations of phenotypes when generating a phenotype map. Hence, while LD can affect pleiotropy *p*-values for few pair of phenotypes, its effect might still be relatively local and can be further weakened during the aggregation and dimension reduction steps of ShinyGPA. In general, this issue can be addressed by preprocessing the GWAS data using LD pruning approaches. However, ideally this issue would be addressed within the statistical framework; hence, we plan to address this issue in our future work.

ShinyGPA is currently publicly available as a part of the R package GPA (http://dongjunchung.github.io/GPA/) providing seamless integration. Overall, we believe that ShinyGPA will be an important tool in the broader context of therapeutic development by providing deeper understanding of the genetic basis of diseases.

## Supporting information

S1 Text Supporting information. (PDF)
