# Supplementary material for: ShinyGPA: An interactive visualization toolkit for investigating pleiotropic architecture using GWAS datasets
Source: PLoS One. 2018 Jan 8;13(1):e0190949. doi: 10.1371/journal.pone.0190949 (PMC5757942; doi:10.1371/journal.pone.0190949)
Supplement: S1 Text — (PDF) [file pone.0190949.s001.pdf]

S1 Text. Supporting Information for "ShinyGPA: An Interactive Visualization Toolkit for Investigating Pleiotropic Architecture Using GWAS Datasets"

Emma Kortemeier[1], Paula S. Ramos[1,2], Kelly J. Hunt[1], Hang J. Kim[3], Gary Hardiman[1,2], and Dongjun Chung[1*]

1 Department of Public Health Sciences, Medical University of South Carolina, Charleston, SC, USA.
2 Department of Medicine, Medical University of South Carolina, Charleston, SC, USA.
3 Department of Mathematical Sciences, University of Cincinnati, Cincinnati, OH, USA.

* Correspondence should be addressed to Dongjun Chung (chungd@musc.edu).

1 Box-Cox Distance Transformation

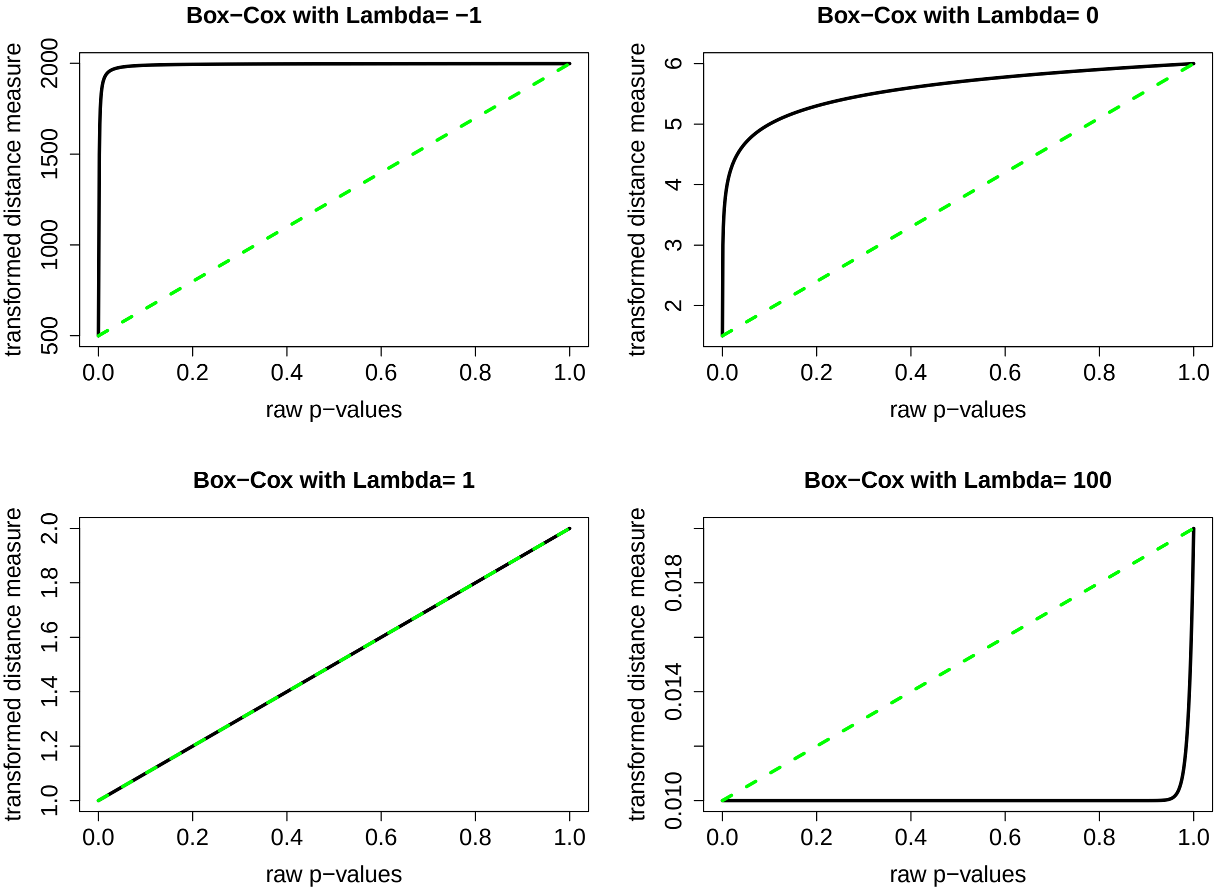

Figure A: Comparisons of raw p-values (y_{ij}) and transformed p-values ($s_{ij}^{(\lambda)}$) in the Box-Cox transformation for different λ values.

2 Description of GWAS Datasets

We used the summary statistics from 12 GWAS datasets as input for ShinyGPA, including

- Neuropsychiatric disorders:
  - Attention deficit-hyperactivity disorder (ADHD), autism spectrum disorder (ASD), bipolar disorder (BPD), major depressive disorder (MDD) and schizophrenia (SCZ).
  - Source: Psychiatric Genomics Consortium (PGC) (`http://www.med.unc.edu/pgc`).
  - References: Cross-Disorder Group of the Psychiatric Genomics Consortium *et al.* (2013); Cross-Disorder Group of the Psychiatric Genomics Consortium (2013).
- Autoimmune disorders:
  - Crohn's disease (CD), ulcerative colitis (UC), and rheumatiod arthritis (RA).
  - Source: International Inflammatory Bowel Disease Genetics Consortium (IIBDGC) (`http://ibdgenetics.org`) for CD and UC; Broad Institute (`http://www.broadinstitute.org/ftp/pub/rheumatoid_arthritis/Stahl_etal_2010NG/`) for RA.
  - References: Franke *et al.* (2010); Anderson *et al.* (2011); Stahl *et al.* (2010).
- Lipid-related phenotypes:
  - High-density lipoprotein (HDL) and type 2 diabetes (T2D).
  - Source: Global Lipids Consortium (GLC) (`http://csg.sph.umich.edu//abecasis/public/lipids2010/`) for HDL; DIAbetesGenetics Replication and Meta-analysis Consortium (DIAGRAM) (`http://diagram-consortium.org`) for T2D.
  - References: Teslovich *et al.* (2010); Morris *et al.* (2012).
- Cardiovascular phenotypes:
  - Coronary artery disease (CAD) and systolic blood pressure (SBP).
  - Source: CARDIoGRAM Consortium (`http://www.cardiogramplusc4d.org/downloads/`) for CAD; International Consortium for Blood Pressure (ICBP) (`http://georgehretlab.org/icbp_088023401234-9812599.html`) for SBP.
  - References: Schunkert *et al.* (2011); International Consortium for Blood Pressure Genome-Wide Association Studies *et al.* (2011).

Table A provides detailed information.

Abbrev.	Phenotype	Source	Num. of Cases	Num. of Controls	Num. of SNPs	Ancestry
ADHD	Attention Deficit/ Hyperactivity Disorder	PGC	1,947 trio; 840	1,947 trio; 688	1,230,535	European
ASD	Autism Spectrum Disorder	PGC	4,788 trio; 161	4,788 trio; 526	1,245,864	European
BPD	Bipolar Disorder	PGC	6,990	4,820	1,233,533	European
MDD	Major Depressive Disorder	PGC	9,227	7,383	1,232,794	European
SCZ	Schizophrenia	PGC	9,379	7,736	1,237,959	European
RA	Rheumatoid Arthritis	Broad Institute	5,539	20,169	2,560,000	European
CD	Crohn's Disease	IIBDGC	3,230	4,829	635,547	European
UC	Ulcerative Colitis	IIBDGC	6,687	19,718	1,100,000	European
HDL	High-Density Lipoprotein	GLC	*	*	187,576	Majority European
T2D	Type 2 Diabetes	DIAGRAM	12,171	56,862	2,500,000	European
CAD	Coronary Artery Disease	CARDIoGRAM	60,801	123,504	5,900,000	European
SBP	Systolic Blood Pressure	ICBP	**	**	2,500,000	European

* 196,476 total cases and controls

** 200,000 total cases and controls

Table A: Details of GWAS datasets for 12 phenotypes.

3 Visualization Results for GWAS Datasets of 12 Phenotypes

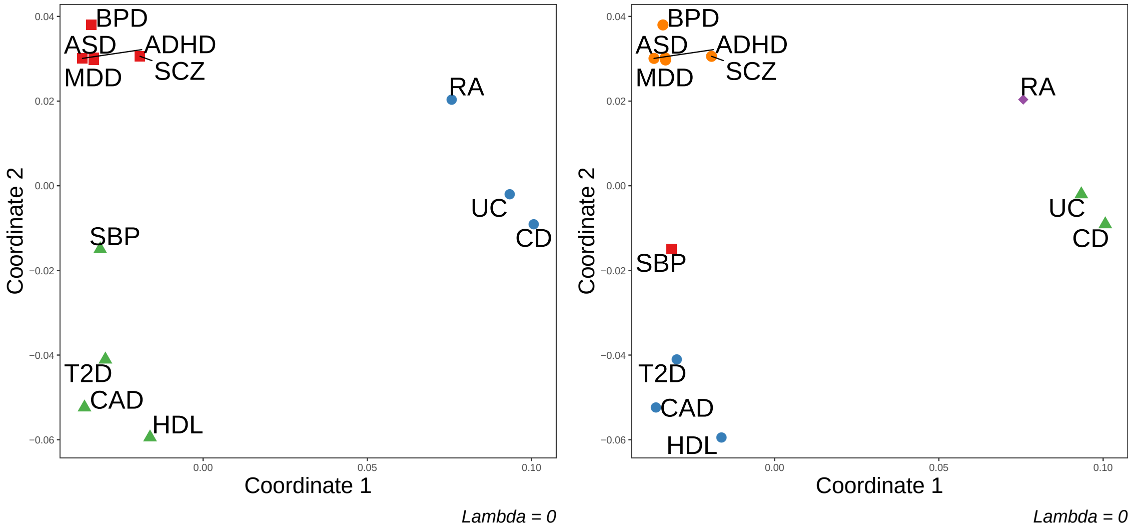

(1) 3 clusters and $\lambda = 0$.

(2) 5 clusters and $\lambda = 0$

Figure B: ShinyGPA plots for the GWAS datasets of 12 phenotypes, showing different clustering options. Different colors and shapes indicate different phenotype clusters.

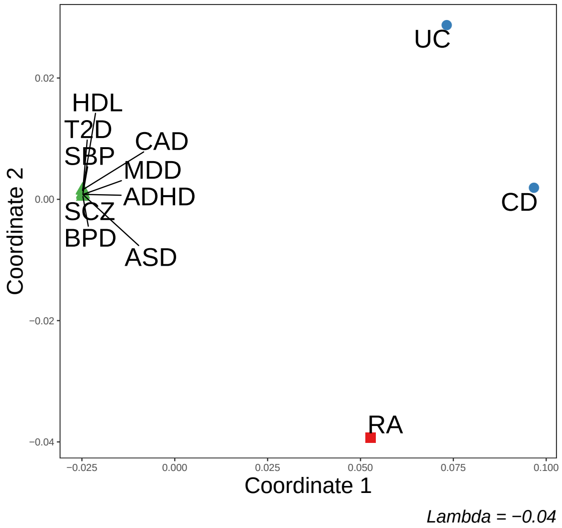

(1) 3 clusters and $\lambda = -0.04$

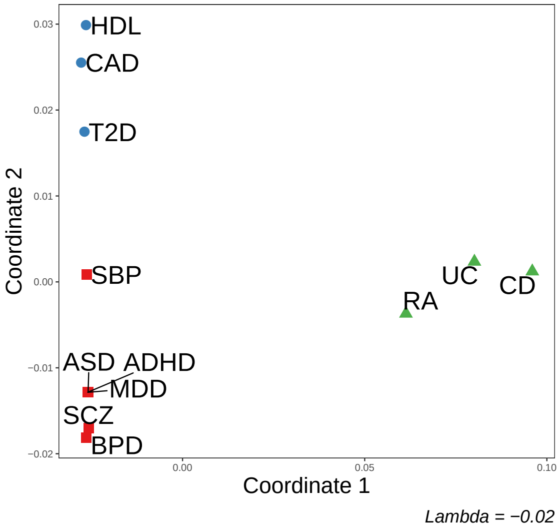

(2) 3 clusters and $\lambda = -0.02$

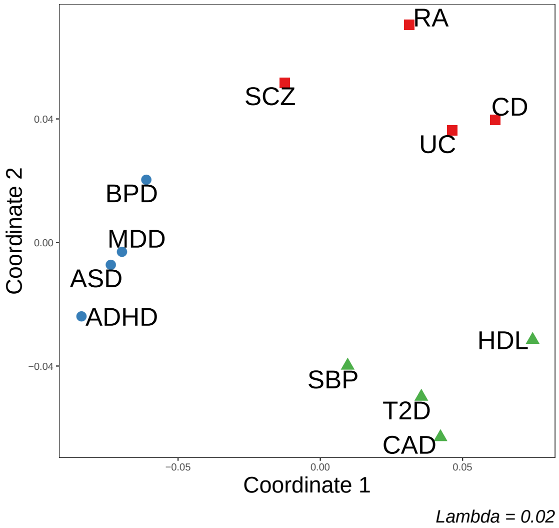

(3) 3 clusters and $\lambda = 0.02$

Figure C: ShinyGPA plots for the GWAS datasets of 12 phenotypes, showing the effect of different λ values. Different colors and shapes indicate different phenotype clusters.

References

Anderson, C. A., Boucher, G., Lees, C. W., Franke, A., D'Amato, M., Taylor, K. D., Lee, J. C., Goyette, P., Imielinski, M., Latiano, A., *et al.* (2011). Meta-analysis identifies 29 additional ulcerative colitis risk loci, increasing the number of confirmed associations to 47. *Nature Genetics*, **43**(3), 246–252.

Cross-Disorder Group of the Psychiatric Genomics Consortium (2013). Identification of risk loci with shared effects on five major psychiatric disorders: a genome-wide analysis. *The Lancet*, **381**(9875), 1371–1379.

Cross-Disorder Group of the Psychiatric Genomics Consortium *et al.* (2013). Genetic relationship between five psychiatric disorders estimated from genome-wide SNPs. *Nature Genetics*, **45**(9), 984–994.

Franke, A., McGovern, D. P., Barrett, J. C., Wang, K., Radford-Smith, G. L., Ahmad, T., Lees, C. W., Balschun, T., Lee, J., Roberts, R., *et al.* (2010). Genome-wide meta-analysis increases to 71 the number of confirmed crohn's disease susceptibility loci. *Nature Genetics*, **42**(12), 1118–1125.

International Consortium for Blood Pressure Genome-Wide Association Studies *et al.* (2011). Genetic variants in novel pathways influence blood pressure and cardiovascular disease risk. *Nature*, **478**(7367), 103–109.

Morris, A. P., Voight, B. F., Teslovich, T. M., Ferreira, T., Segre, A. V., Steinthorsdottir, V., Strawbridge, R. J., Khan, H., Grallert, H., Mahajan, A., *et al.* (2012). Large-scale association analysis provides insights into the genetic architecture and pathophysiology of type 2 diabetes. *Nature Genetics*, **44**(9), 981–990.

Schunkert, H., König, I. R., Kathiresan, S., Reilly, M. P., Assimes, T. L., Holm, H., Preuss, M., Stewart, A. F., Barbalic, M., Gieger, C., *et al.* (2011). Large-scale association analysis identifies 13 new susceptibility loci for coronary artery disease. *Nature Genetics*, **43**(4), 333–338.

Stahl, E. A., Raychaudhuri, S., Remmers, E. F., Xie, G., Eyre, S., Thomson, B. P., Li, Y., Kurreeman, F. A., Zhernakova, A., Hinks, A., *et al.* (2010). Genome-wide association study meta-analysis identifies seven new rheumatoid arthritis risk loci. *Nature Genetics*, **42**(6), 508–514.

Teslovich, T. M., Musunuru, K., Smith, A. V., Edmondson, A. C., Stylianou, I. M., Koseki, M., Pirruccello, J. P., Ripatti, S., Chasman, D. I., Willer, C. J., *et al.* (2010). Biological, clinical and population relevance of 95 loci for blood lipids. *Nature*, **466**(7307), 707–713.
